# Development and Evaluation of the Reliability of a Semi-Quantitative Food Frequency Questionnaire to Assess the Intake in the Serbian Population

**DOI:** 10.3390/nu16152490

**Published:** 2024-07-31

**Authors:** Silvia Garcia, Bojana Vidović, Alexandra Tijerina, Josep A. Tur, Cristina Bouzas

**Affiliations:** 1 Research Group on Community Nutrition and Oxidative Stress, University of the Balearic Islands—IUNICS, 07122 Palma de Mallorca, Spain; 2 Health Institute of the Balearic Islands (IDISBA), 07120 Palma de Mallorca, Spain; 3 CIBER Physiopathology of Obesity and Nutrition (CIBEROBN), Institute of Health Carlos III (ISCIII), 28029 Madrid, Spain; 4 Department of Bromatology, Faculty of Pharmacy, University of Belgrade, 11221 Belgrade, Serbia; bojana.vidovic@pharmacy.bg.ac.rs; 5 Faculty of Public Health and Nutrition, Autonomous University of Nuevo Leon, Monterrey 64460, Mexico; alexandra.tijerinas@uanl.mx

**Keywords:** validation, food frequency questionnaire, 24 h recall, nutrients, Serbia

## Abstract

Assessing dietary habits with validated questionnaires is crucial for achieving reliable results in health research. The aim of this study was the development and validation of a semi-quantitative food frequency questionnaire (FFQ) in an 18–30-year-old Serbian population. A total of 93 participants completed the FFQ and three 24 h dietary recalls (24 hR). Pearson and intraclass correlations between FFQ and 24 hR intakes were assessed and were de-attenuated and energy-adjusted. Bland–Altman plots were generated, and quintiles of energy, water, fiber, and macronutrient intake were analyzed with contingency tables. Adjustments for sex differences were included. The validity of the FFQ varied, with significant correlations for energy, carbohydrates, cholesterol, and vitamin B_12_, vitamin C and vitamin D. Misclassification rates were generally low. Bland–Altman plots indicated good agreement between methods. It can be concluded that the validated FFQ is a promising tool for dietary assessment in the Serbian population. Results for several nutrients align closely with previous studies. The new FFQ is a useful tool for dietary assessment in the Serbian population.

## 1. Introduction

Assessing dietary habits and nutritional intake in a population is fundamental for understanding the relationship between diet and health outcomes [1]. One valuable tool for this task is the food frequency questionnaire (FFQ) [2]. This questionnaire allows researchers to gather comprehensive data on an individual’s long-term eating habits by asking how often they consume specific foods and beverages over a defined period and enabling them to analyze dietary patterns and nutrient intakes [3].

Additionally, validated food questionnaires play a critical role in epidemiological studies investigating the links between diet and various health outcomes, including chronic diseases such as obesity, diabetes, and cardiovascular disease [1]. It is crucial to quantify and rectify measurement errors in dietary assessment tools like the FFQ, which is particularly important for establishing the relative accuracy of the instrument, often using multiple 24 h dietary recalls (24 hR) as the gold standard [4]. Reliable data on dietary intake can help identify risk factors and potential areas for intervention, ultimately contributing to the development of evidence-based dietary recommendations and preventive strategies [1]. While FFQs have been widely employed in nutritional research worldwide, there remains a noticeable gap in their utilization and validation within the young adult population of Serbia.

Existing studies on dietary intake in Serbia primarily rely on data from the Serbian National Health Survey [5,6], last updated in 2019 [7], or analysis of the nutrient content of specific food items available in the Serbian market [8,9,10]. These approaches, while informative, do not capture the comprehensive dietary habits of the population.

Recent validations of FFQs in Serbia have been conducted for specific nutrients, such as vitamin D [11] and folic acid [12], and for specific populations, such as young women and women of reproductive age, respectively. A recent study also employed an FFQ to determine the intake of milk and dairy products [13]. However, a validated FFQ designed to assess the overall nutritional quality of the standard Serbian diet is absent from the literature.

Dietary habits and cultural influences on food consumption vary significantly across different countries [14,15,16]. Each region has its unique culinary traditions, food availability, and social norms regarding diet, which impact the types of foods consumed and their preparation methods, as is also the case in Serbia [6]. Therefore, creating and validating country-specific FFQs is essential to reflect these unique dietary patterns. Non-specific questionnaires can lead to data that do not accurately represent the population’s true dietary intake, leading to misguided health recommendations and policies. Personalized FFQs ensure that the collected data are relevant and reliable, enabling researchers to make precise assessments of dietary habits and their impacts on health [17].

Understanding dietary habits and nutritional intake is crucial not only for public health initiatives but also for personalized nutrition approaches. The development and validation of an FFQ tailored to specific populations are essential for capturing the nuances of dietary behaviors that vary with age, cultural background, and socioeconomic status, which are also currently influenced by increased food availability, changes in lifestyle, and the transition toward a more Western and processed diet [18].

Additionally, young adults are at a life stage where dietary habits can have long-lasting implications on their health [19], making accurate dietary assessment tools indispensable for both research and practical dietary guidance. By validating an FFQ specifically for this demographic, researchers can ensure more precise data collection, leading to better-informed public health policies and targeted nutritional interventions. This study aims to fill the gap in the current literature by providing a robust, culturally appropriate FFQ for young adults in Serbia, thereby enhancing the accuracy of dietary assessments and contributing to the global understanding of diet-health relationships.

Having an FFQ validated in a Serbian population can contribute valuable insights to the field of nutrition and public health in Serbia, fostering evidence-based strategies for promoting healthier dietary choices and improving overall well-being.

Therefore, the purpose of this study is the development and validation of a semi-quantitative FFQ in an 18–30-year-old Serbian population.

## 2. Methodology

### 2.1. Participants, Recruitment, and Ethics

**Figure 1:**
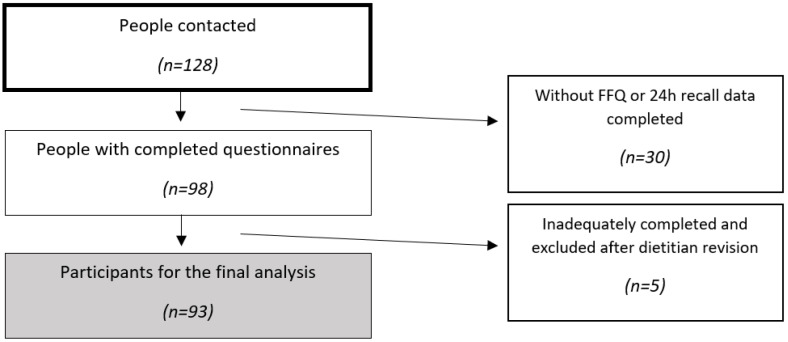
Participant inclusion flow-chart.

In this study, a total of 128 participants were enrolled, of whom 98 completed the required questionnaires. All questionnaires were reviewed by a certified dietitian–nutritionist, and participants with inadequately completed questionnaires were excluded. Excluded participants accounted for 27.3% of the sample, primarily due to issues such as duplicative entries in the FFQ or omissions in reporting water consumption in the 24 hR, among other completion errors. Consequently, the final analysis included a sample of 93 participants. A flow-chart of participant inclusion can be seen in Figure 1. The inclusion criteria stipulated that participants had to be Serbian citizens and 18 to 30 years old; it included both men and women. We focused on adults aged 18–30 because individuals under 18 typically have limited control over their diets, while those over 30 may have established habits influenced by different generations. The 18–30 age range captures critical years for establishing and adapting dietary patterns. This group includes both those in transitional phases (e.g., living with parents, studying) and those with more stable habits (e.g., working, living independently), making it representative of current generational trends. Exclusion criteria were applied to individuals with preexisting medical conditions, those currently taking medications, and pregnant or lactating women. Dietary assessments and anthropometric measurements for the recruitment process were conducted at the Department of Bromatology (Faculty of Pharmacy, University of Belgrade). Prior to participation, informed written consent was provided by all participants. This study was conducted according to the Declaration of Helsinki and approved by the Ethics and Research Committee of the Faculty of Pharmacy, University of Belgrade (Reference Number: 1770/2, Date: 6 July 2023).

### 2.2. Food Frequency Questionnaire Development

The FFQ used in this study was based on already validated questionnaires [20,21,22,23]. To ensure their relevance to the dietary patterns of the Serbian population, an adaptation process was undertaken. Each food item featured in the FFQ was reviewed and changed, if necessary, according to usual Serbian dietary habits. The goal was to identify and replace foods that were not representative of the Serbian context with suitable alternatives found within this country’s dietary landscape. An initial trial involving a small sample of 12 participants was conducted to assess the consistency of responses. The feedback and results from this pilot study were used to refine the FFQ and the methodology, ensuring that the definitive study is more accurate and representative.

The FFQ was designed to be semi-quantitative to assess usual dietary intake in an 18–30-year-old Serbian population. A total number of 149 food items and 9 food groups (dairies, animal foods, vegetables, fruits, cereals and legumes, fats and oils, pastry, miscellaneous, and beverages) were listed in the developed FFQ. A regular portion size was established, and consumption frequencies were set ranging from “never or almost never” to “≥6 times/day”. Those 9 FFQ groups and the items included in each group, translated from the Serbian questionnaire to English, are shown in Table 1. Original Serbian questionnaire is shown as Supplementary Table S1.

### 2.3. Food Frequency Questionnaire Validation

Validation of the FFQ was performed between June 2023 and September 2023. A total of *n* = 93 18–30-year-old participants completed one FFQ and three 24 h recalls (24 hR used as a reference method).

Trained dietitians performed the FFQs during personal interviews. During the same interview, the three-day 24 hR was explained and sent by email to be self-answered at home and sent back when finished. Then, they were revised by a dietitian. Two 24 hR were answered to evaluate intakes during weekdays (e.g., Monday to Thursday) and one during a weekend day (e.g., Friday, Saturday, or Sunday). The three 24 hR also included information about the time of consumption, place of consumption, kind of food consumed and serving size, which could be expressed in common measures (cup, piece, teaspoon, tablespoon, or slice) or by weight in grams (g) and volume in milliliters (mL).

Energy, macronutrient, and micronutrient intake calculated from the FFQ were determined by multiplying the consumption frequency with the nutrient content of the specified portion size for each food item. Daily intake values were calculated by dividing the results by a standard week of 7 days.

The information related to the 24 hR was analyzed in EvalFINUT^®^ version 2.0., which is based on information from BEDCA (Base de Datos Española de Composición de Alimentos) and USDA (United States Department of Agriculture) food databases [24]. This program allows the conversion of food data into dietary intakes of macronutrients and micronutrients. The means of the three 24 hR were used as a reference for the validation study. The results of dietary intakes were reported based on daily consumption in kilocalories (kcal) for energy and in grams (g), milligrams (mg), or micrograms (μg) for macronutrients and micronutrients.

### 2.4. General Data and Anthropometric Measurements

Information related to sociodemographic characteristics such as sex, date of birth, city of birth, city of residence, level of education, job status as well as the dietary pattern (omnivorous, vegetarian, or vegan), alcohol and smoking patterns, allergies or food intolerances, supplements and drugs consumption, and physical activity level were self-reported.

Anthropometric measurements (weight, height, waist, hip circumference, and tricep fold) were obtained during the first personal interview. Weight was measured with participants wearing light clothes and no shoes using a Tanita body composition monitor BC-587. Height was measured to the nearest millimeter, with the participant’s head maintained in the Frankfurt Horizontal Plane using a portable KaWe PERSON-CHECK person measuring device. Waist circumference was measured halfway between the last rib and the iliac crest with anthropometric tape with the subjects standing upright. Hip circumference was measured at the widest point of the hips, also using anthropometric tape, and tricep fold was measured using a Holtain Tanner/Whitehouse Skinfold Caliper (Holtain Ltd., Crosswell, Crymych, UK).

All measurements were measured twice, and the average value of each one of them was used in the analysis.

### 2.5. Statistics

Analyses were performed with SPSS statistical software package version 25.0 (SPPS Inc., Chicago, IL, USA). Data related to demographic characteristics were shown as mean and standard deviation (SD); except for prevalence data, which were expressed as sample size and percentage. After outlier removal, the Kolmogorov–Smirnov test was used to analyze the normality of dietary data, resulting in a non-normal distribution. FFQ validation was carried out via the log_10_ transformation of data to improve normality. A Pearson correlation coefficient was calculated for both logs: transformed intakes and energy-adjusted nutrient intakes based on the residual method [23,25]. Intraclass correlation coefficients (ICCs) were also calculated, serving as valuable indicators for evaluating the reliability of dietary methods within the validity assessment of the FFQs [26]. Both correlation coefficients were multiplied using the following de-attenuation factor, √1 + (S2w/S2b)/*n*, to account for within-individual error in 24 hR. “S2w” was the within-person variance, “S2b” was the between-person variance, and “*n*” was the number of replicate measurements of dietary recalls (*n* = 3). Variances S2w and S2b were determined using the random effects model adjusted by sex, with dietary intakes from 24 hR as the dependent variable and each participant’s identification number as the independent variable [23,27,28]. Quintiles of energy, water, fiber, and macronutrient intake (protein, carbohydrates, and fat) were computed for both FFQ and 24 hR to assess the misclassification of participants concerning intake distributions analyzed via contingency tables. Bland–Altman plots for energy, water, fiber, and macronutrient values were generated to illustrate the agreement between methods. These plots represent the difference in dietary intakes between methods (FFQ2–24 hR) against the average intake ([FFQ2 + 24 hR]/2). The plots display lines for the average difference in intakes and the limits of agreement, defined as ±1.96 standard deviations from the average difference in intake.

## 3. Results

A total number of 93 Serbian participants (<30 years old) were included in the validation study after completing one FFQ and three 24 hR. Descriptive data of the study participants are given in Table 2. The total sample consisted of 39 men and 54 women. The mean age was 24.3 (SD = 2.6) years. The mean weight was 71.7 kg (SD = 18.5), and the mean height was 1.72 m (SD = 0.2). BMI was 23.6 kg/m^2^ (SD = 4.1), with 54.8% of the population categorized as having a normal weight.

Table 3 shows intakes of energy, macronutrients, and micronutrients, which were estimated according to both questionnaires. Means and standard deviations were calculated. Mean intakes in FFQ were higher than those from 24 hR, except for polyunsaturated fats, cholesterol, and vitamin D.

Table 4 shows the relative validity of the FFQ against the average of three 24 hR for intakes of energy, water, macronutrients, fiber, cholesterol, minerals, and vitamins, calculated with Pearson correlation coefficients and ICC.

Pearson coefficients of correlation for the validity of FFQ in relation to 24 hR presented a wide variation, going from 0.05 to 0.32 and from −0.08 to 0.33, for energy-unadjusted and adjusted data, respectively. Pearson correlation was statistically significant for energy (0.24). Energy-adjusted correlation coefficients showing statistical significance (*p* < 0.05) were the ones referring to carbohydrates (0.23), cholesterol (0.33), vitamin B_12_ (0.23), vitamin C (0.22), and vitamin D (0.24). Sodium was statistically significant when unadjusted but lost its significance when adjusting for energy intake.

ICCs ranged from 0.01 to 0.84 and from −0.17 to 0.50 for energy-unadjusted and adjusted data, respectively. ICCs were statistically significant for energy (0.13). ICCs (energy-adjusted) showing statistical significance (*p* < 0.05) were good for carbohydrates (0.37), cholesterol (0.50), vitamin B_12_ (0.37), vitamin D (0.36), and vitamin C (0.49).

The percentage of misclassification of energy and macronutrients is shown in Table 5. Overall, all nutrients showed lower than 5% misclassification, except for protein (5.3%), fiber (5,3%), and fat (6.4%). Participants were correctly classified in the same or adjacent quintile for energy and macronutrient intake in 49.4–66.6% (mean 58%). The nutrient with the highest concordance was carbohydrates (66.6%). The lowest concordance was for fiber (49.4%). Energy (57.1%), water (55.9%), protein (58.1%), and fat (58.1%) were in between the highest and the lowest concordances.

**Figure 2:**
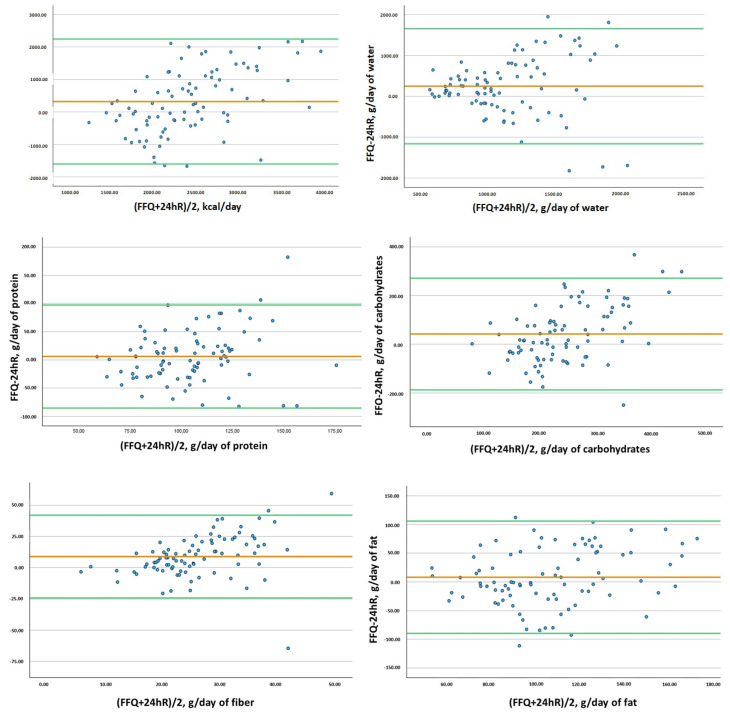
Bland–Altman plots showing the difference in intakes and the mean intakes of energy, protein, fat, carbohydrates, water, and fiber, estimated by the developed FFQ and the average of three 24 hR. Orange lines are mean differences in intakes, and green lines are the upper and lower limits (95% CI) of agreement between methods. FFQ, food frequency questionnaire; 24 hR, 24 h dietary recall.

Bland–Altman plots are shown in Figure 2 to visualize dietary intakes of energy, water, protein, carbohydrates, fiber, and fat. All mean difference values of intake were close to zero, indicating that the average values for both methods were similar. The vast majority of measurements fall within the range defined by the limits of agreement, indicating great agreement between both methods. Variability appears to be consistent across the range of values as the scatter points are not only located below or above the mean line but are distributed around the mean difference and do not follow any trend.

## 4. Discussion

A semi-quantitative FFQ containing 149 items was adapted and validated for application to an 18–30-year-old Serbian population. Validation was conducted for the following 16 dietary items: energy intake (kcal), water (mL), protein (g), carbohydrates (g), fiber (g), fat (g), saturated fat (g), monounsaturated fat (g), polyunsaturated fat (g), cholesterol (mg), calcium (mg), sodium (mg), phosphorous (mg), vitamin B_12_ (µg), vitamin C (mg), and vitamin D (µg).

Only two FFQ validation studies have been carried out for a Serbian population, focused on young women and specific nutrients [11,12]. Both of the studies only analyzed macronutrients, folate [12], or vitamin D and calcium [11]. However, a few comparisons can be made between them and our current study. The Pearson correlation coefficient was calculated in one of the mentioned studies [12], and the results were higher for carbohydrates (0.52) than ours (0.23). Cross-classification in the two studies mentioned was carried out with quartiles and only for one specific nutrient [11,12]; therefore, comparisons with our results are difficult because we used quintiles and did so for energy, water, fiber, and macronutrients. In our case, when examining the quintiles of intake, most of the nutrients exhibited less than 5% misclassification between the FFQ and the three 24 hR, showing the overall reliability of the dietary instrument. Bland–Altman plots were shown in three of the studies, all three showed good agreement between methods.

FFQ validations have also been carried out in other countries and for different populations [26,27,29,30,31,32]. The present FFQ was developed based on already validated questionnaires [21,22,23], and the methodology employed was replicated from them, along with another validation conducted in a Mexican population [27]. The Pearson correlation-corrected coefficients were similar between those studies but showed better or worse correlations depending on the study and the nutrient observed. It is important to assume that disparities between correlation coefficients are common and change across food groups and nutrients, depending on the validation performed [33].

A previous study focused on a Spanish population [21] showed Pearson correlations of 0.31 for carbohydrates, 0.48 for vitamin B_12_, 0.62 for vitamin C, and 0.51 for vitamin D, but cholesterol was not calculated. Another Spanish study [22] showed values of 0.40 for carbohydrates, 0.55 for cholesterol, and 0.52 for vitamin C, without calculations for vitamin D and vitamin B_12_. A third study on an older Spanish population [23] showed values of 0.56 for carbohydrates, 0.23 for cholesterol, 0.68 for vitamin C, 0.24 for vitamin D, and no calculations of vitamin B_12_. Finally, A Mexican survey [27] showed values of 0.41 for carbohydrates, 0.42 for cholesterol, 0.43 for vitamin B12, 0.60 for vitamin D and no calculations for vitamin C. The current results using Pearson correlation coefficients were higher for cholesterol (0.33) compared to a previous one in Spain [17] but lower for carbohydrates (0.23) compared to the four studies mentioned. Vitamin B_12_ was also lower in our study (0.23) compared to two other previous studies [21,27] that calculated it, and the same happened for vitamin C (0.22) [21,22,23] and vitamin D (0.24) [21,23,27].

ICCs were only calculated in the two previous studies focused on Spanish [17] and Mexican populations [21]. In our validation, ICCs were good for carbohydrates (0.37), being lower when compared to those found in Spain [17] (0.80) and Mexico [21] (0.65). Our ICC for cholesterol (0.50) was higher compared to the Mexican population [21] (0.33) but lower compared to the Spanish survey [17] (0.72). Vitamin B_12_ was only calculated for Mexicans [21] (0.72) and showed higher values than ours (0.37), and vitamin C (0.36), which was only calculated in a Spanish population [17], and also presented higher values (0.87). Regarding vitamin D, our ICC value (0.49) was lower than those found in a Mexican population [21] (0.72) but higher compared to that found in a Spanish population [17] (0.39).

FFQ validations have been carried out in some countries of the Balkan region as well, mainly in Croatia [34,35,36,37]. The methodologies used in Balkan validations differ slightly among themselves and from our validation. For example, in a Greek FFQ validation, energy and macronutrient intake showed validity; however, Kendall’s tau-b coefficients were used [37] instead of Pearson correlations and the ICCs; therefore, the results are not comparable. However, Bland–Altmann plots were calculated, showing worse results than those found in our study because the limits of agreement were relatively high [37], while ours showed good agreement between methods. A validation study carried out using Croatian adults used Pearson correlation due to its validation, showing values of 0.33 for energy, 0.42 for carbohydrates, 0.44 for cholesterol, 0.25 for vitamin C, and no calculations for vitamin B_12_ or D [35]. All values were slightly higher than those found in the present validation study, but the study did not use ICCs or Bland–Altmann plots afterward to assess consistency or agreement, respectively. Two more recent FFQ validations were carried out in Croatia for young people [34] and adolescents [36]. The FFQ validated for young people assessed that the validated FFQ was a reliable method but used Cronbach’s alpha coefficient for the validation [34], which, again, is not directly comparable to our results. Bland–Altmann plots were calculated between FFQ1 and FFQ2 [34] but not between FFQ and 24 hR, as in our validation study. The FFQ validated for adolescents showed significant correlations for energy intake, carbohydrates, cholesterol, and vitamin C, like in our validation, and vitamins B12 or D were not calculated; however, the calculations were conducted with Spearman’s correlation coefficients, so the results are not specifically comparable [36]. Bland–Altman analysis was carried out and, as in our results, showed good agreement with all macronutrients [36]. Cross-classification was also calculated in this last study and revealed that a mean of 76.5% of adolescents were classified in the same or adjacent quartile [36], in comparison with our 58%. However, our cross-classification was performed with quintiles instead of quartiles, which makes comparisons difficult. The Balkan region, which now comprises many countries, was once part of the former Yugoslavia, resulting in shared cultural and dietary customs across these nations [38]. Given these similarities, it is conceivable that a future initiative could involve creating a unified FFQ for the entire region. This validation, along with the others conducted in the Balkan region, could be the first step toward the creation and validation of a unified Balkan FFQ.

In this validation, both correlation coefficients were de-attenuated to account for within-individual error in 24 hR. The de-attenuation procedure was based on previously published literature [39] and was used in other FFQ validations [21,22,23,27]. Additionally, an energy adjustment for nutrients was performed using the residual method based on a linear regression model [28]. Correlation coefficient-adjusted values are the ones being compared with the rest of the validation studies mentioned. This last adjustment was necessary because the estimated intake of energy and the majority of macronutrients and micronutrients from the FFQ was significantly higher than the intake recorded when using the reference method (24 hR).

This trend is frequently observed in research focused on the creation and validation of FFQs across different populations [27,40,41,42,43]. A recent study on the validation of an FFQ among adult Saudi subjects in Jeddah found that the FFQ used generally overestimated the intake of most nutrients compared to 24 hR, except for fat and cholesterol [41]. Similar to the findings of the adult Saudi study, our validation also revealed some exceptions, such as polyunsaturated fat, cholesterol, and vitamin D being lower in the FFQs compared to the 24 hR.

A systematic review evaluating dietary assessment methods in pregnant women revealed that FFQs often reported higher nutrient intakes than 24 hR, highlighting the tendency of FFQs to overestimate intake [40]. Similarly, an investigation into energy and nutrient intake estimates using FFQs versus weighed food records showed that the FFQs consistently reported higher intake values. This pattern was attributed to standard portion sizes and the broader range of foods considered in FFQs compared to the more precise recording in weighed food records [42].

Overreporting dietary intake can be due to misestimations of portion sizes and consumption frequencies. Foods that are consumed regularly are often overreported, while those with a negative health perception are underreported [37,44,45,46,47]. Furthermore, individual memory biases tend to cause overreporting of minor intakes and underreporting of major ones in the FFQ. This issue is also common when using 24 hR to assess dietary intake from the previous day [48,49].

Several validation studies are specific in terms of sex, like the ones conducted in Serbia [11,12] or the one carried out in Mexico [27], which were only carried out using female populations. In our validation, we included female and male adults, which creates the need to consider both for the analysis. The absence of sex consideration in FFQ development leads to a more significant discrepancy in assessing dietary intake among women compared to men [50]. To incorporate sex differences, variances calculated using the random effects model were adjusted by sex.

Findings from this validation study highlight the importance of comprehensive data collection and accurate methodologies in dietary assessments. The sample size varies widely across validation studies, ranging from 20 to 530 participants according to a recent systematic review [33], all considered acceptable for validation if the methodology is carefully adhered to. In our validation study, several nutrients, such as carbohydrates, cholesterol, vitamin B_12_, vitamin C, and vitamin D, exhibited a good level of agreement, demonstrating the reliability of the dietary instrument. It is common to see how almost all the validation studies found some weak correlations or exceptions for some food groups and nutrients when validating but were determined as valid and reproducible questionnaires [33,51,52].

This study reflects the structure of the Serbian population in terms of overweight and obesity. According to the EUROSTAT and Serbian data [53,54], 53.6% of the Serbian population has excess body weight, with 24.1% of the 18–24-year-old Serbian population and 42.1% of the 25–34-year-old Serbian population comprising 33.3% of the 18–30-year-old Serbian people that have excess of body weight, which is very close to the percentage of the recruited participants found to have excess body weight in this study (35%). It could be thought that being under/overweight could affect the results; however, the FFQ was compared with 3 × 24 hR for each participant; therefore, this under/overweight condition did not affect the obtained results.

This study provides valuable insights that contribute to the development and refinement of FFQs for different populations. The positive results reinforce the importance of accurate data gathering and underscore the utility of FFQs in nutritional studies, allowing continuous improvement and adaptation of these essential tools.

## 5. Strengths and Limitations of the Study

This validation contributes to the field of nutrition and public health in Serbia, enabling accurate and reliable dietary assessment studies within the country. The inclusion of both females and males in the validation and the adjustment of the analysis according to sex can be considered the first strength. The de-attenuation of the correlation coefficients and the energy adjustment are the second and third strengths, as they account for within-individual error in 24 hR and for the overestimation of dietary intake, respectively. The use of the 24 hR to compare with the FFQ is another strength since 24 hR is often used as the gold standard. The analysis of a total of 16 dietary items, which is a good number of items, gives a broader image of the whole diet and, thus, gives more information to the validation procedure.

This study has some limitations. It focuses on young and middle-aged adults, which is greatly representative of the population; however, this focus does not allow for extrapolation to children or older people. Another limitation is the sample size, which is not small and aligns with the samples used for validation studies; however, it could have been higher if everyone included in the study had answered the questionnaires. The reliance on nutritional databases from Spain and the USA is another limitation. While these databases provide accurate and high-quality nutritional information, they may not fully capture regional variations in terms of food composition and nutritional values. The fact that the recruitment process was performed at the Department of Bromatology could limit the participants to an academic population. Finally, data collection was only carried out for three months, and reproducibility was not evaluated.

Future research plans will focus on expanding the applicability of the newly validated FFQ beyond young and middle-aged adults to include broader age groups, such as children and older people, to enhance its generalizability across the Serbian population. Efforts will also be directed towards increasing the sample size to ensure robustness and reliability in the dietary assessments. Longitudinal studies could be conducted to evaluate the reproducibility of the FFQ over time, providing insights into its stability and suitability for longitudinal dietary research. Additionally, collaborations with diverse community settings beyond academic institutions will be pursued to ensure the representation of various socioeconomic and cultural backgrounds, thereby enriching the applicability and relevance of the FFQ in real-world nutritional studies in Serbia and beyond. Future research could also focus on developing and validating a unified FFQ for the Balkan region, using shared cultural and dietary customs from the former Yugoslavia. These future directions aim to further strengthen the utility and effectiveness of dietary assessment tools in promoting public health and nutrition research.

## 6. Conclusions

The new FFQ demonstrates its utility as a robust tool for dietary assessment in the Serbian population. Statistical analyses revealed significant correlations for key nutrients, including energy intake, carbohydrates, cholesterol, vitamin B_12_, vitamin D, and vitamin C. Bland–Altmann plots showed great agreement between the FFQ and the three 24 hR. These findings corroborate the validity and reliability of the FFQ, aligning closely with previous studies. Overall, this validation study substantively contributes to evidence-based health and nutrition research, offering researchers a validated tool to refine dietary assessments in Serbia.

## Figures and Tables

**Table 1:** FFQ food groups and items included in each group.

I. MILK AND DAIRY PRODUCTS	II. EGGS, MEAT, FISH (1 SERVING OR 100–150 g, EXCEPT WHEN ANOTHER AMOUNT IS INDICATED)	III. VEGETABLES (1 SERBING OR 200 g, EXCEPT WHEN ANOTHER AMOUNT IS INDICATED)
Whole milk (1 cup, 200 mL) Semi-skimmed milk (1 cup, 200 mL) Skimmed milk (1 cup, 200 mL) Plant-based milk substitutes (soy, oat …) (1 cup, 200 mL) Cream (1 tablespoon) Sour cream (1/2 cup) Milkshake (1 glass, 200 mL) Yogurt (1 glass, 125 mL) Low-fat yogurt (1 glass, 125 mL) Fruit yogurt (1 glass) Fresh, cottage cheese (1/2 cup) Cream cheese (1 serving 25 g) Hard/semi-hard cheese (50 g) White cheese (50 g) Cream, pudding (1 piece, 130 mL) Ice cream (1 piece)	17. Egg (1) 18. Chicken or turkey, with skin (1 serving or piece) 19. Chicken or turkey, without skin (1 serving or piece) 20. Veal/beef (1 serving) 21. Pork (1 serving) 22. Lamb (1 serving) 23. Rabbit (1 serving) 24. Liver (1 serving) 25. Ther offal (1 serving) 26. Prosciutto (1 slice, 30 g) 27. Ham (1 slice, 30 g) 28. Processed meats: sausages, hot dogs (50 g) 29. Pâté (25 g) 30. Hamburger (1 piece), meatballs (3 pieces) 31. Bacon, pancetta (50 g) 32. White fish: hake, sea bream, redfish, sole, etc. (1 piece or serving) 33. Blue fish: sardines, tuna, mackerel, salmon (1 piece or serving) 34. Salted fish: cod, smoked carp (1 serving, 60 g) 35. Oysters, clams, mussels, and similar (6 pieces) 36. Squid, octopus (1 serving, 200 g) 37. Shrimps (4–5 pieces, 200 g) 38. Canned fish in brine: sardines, tuna… (1 can) 39. Canned fish in oil: sardines, tuna… (1 can)	40. Chard, spinach 41. Cabbage, cauliflower, broccoli 42. Lettuce (100 g) 43. Fresh tomato (1p iece,150 g) 44. Carrot, pumpkin (100 g) 45. Green beans (green, yellow) 46. Eggplant, zucchini, cucumbers 47. Bell pepper (150 g) 48. Asparagus 49. Tomato juice (1 glass, 200 g) 50. Other vegetables (artichoke, leek, celery) 51. Red onion (half a head, 50 g) 52. Garlic (1 clove) 53. Parsley, thyme, oregano, etc. (1 pinch) 54. Frozen fries 55. Homemade fries (1 serving, 150 g) 56. Steamed or boiled potatoes 57. Champignons, chanterelles, other mushrooms
IV. FRUITS (1 PIECE OR SERVING)	V. CEREALS AND LEGUMES (1 SERVING)	VI. FATS AND OILS (1 TABLESPOON OR INDIVIDUAL PORTION FOR FRYING, SPREADING, FOR SALADS, TOTAL)
58. Orange (1 piece), grapefruit (1 piece) or tangerines (2 pieces) 59. Banana (1 piece) 60. Apple, pear (1 piece) 61. Strawberries (6 pieces, 1 dessert plate) 62. Cherries, sour cherries, plums (1 handful/dessert plate) 63. Peach, nectarine, apricot (1 piece) 64. Watermelon (1 slice, 200–250 g) 65. Melon (1 piece, 200–250 g) 66. Kiwi (1 piece, 100 g) 67. Grapes (1/2 bunch) 68. Tropical fruit: pineapple, papaya, mango (150–200 g) 69. Berries: blueberries, blackberries, raspberries (100 g) 70. Avocado (150–200 g) 71. Olives (10 pieces) 72. Candied fruit (2 pieces) 73. Dates, dried figs, raisins, prunes (50 g) 74. Pistachios (30 g) 75. Almonds (30 g) 76. Walnuts (30 g) 77. Other nuts (30 g) 78. How many times a week do you have fruit as a dessert?	79. Lentils (1 plate, 150 g) 80. Beans (1 plate, 150 g) 81. Chickpeas (1 plate, 150 g) 82. Peas (1 plate, 150 g) 83. White wheat bread (1 slice, 75 g) 84. Whole wheat bread (1 slice, 75 g) 85. Bread from other grains: rye, corn, tortillas, etc. (1 slice, 75 g) 86. Instant breakfast cereals (30 g) 87. Whole grain cereals; muesli, oatmeal (30 g) 88. Rice (60 g) 89. Brown rice (60 g) 90. Pasta: noodles, macaroni, spaghetti (60 g) 91. Whole grain pasta: noodles, macaroni, spaghetti (60 g) 92. Pizza (1 slice, 200 g) How often do you consume? 93. Fried food at home 94. Fried food in a restaurant	95. Olive oil (1 tablespoon) 96. Virgin or extra virgin olive oil (1 tablespoon) 97. Pomace olive oil (1 tablespoon) 98. Corn oil (1 tablespoon) 99. Sunflower oil (1 tablespoon) 100. Soybean oil (1 tablespoon) 101. Blended vegetable oils (1 tablespoon) 102. Margarine (portion, 12 g) 103. Salted butter (portion, 12 g) 104. Unsalted butter (portion, 12 g) 105. Lard (10 g) 106. Brand of olive oil you usually use:
VII. SWEET PASTRIES AND CONFECTIONERY	VIII. MISCELLANEOUS	IX. BEVERAGES
107. Plazma biscuits (6 pieces, 50 g) 108. Whole grain biscuits (4–6 pieces, 50 g) 109. Chocolate biscuits (4 pieces, 50 g) 110. Homemade cake (50 g) 111. Croissant (1 piece, 50 g) 112. Donut (1 piece) 113. Muffins (1–2 pieces) 114. Homemade cake (1 piece) 115. Fritters, fried dough (1 serving, 100 g) 116. Chocolate and confectionery products (30 g) 117. Cocoa (1 tablespoon) 118. Bonzita (1 piece) 119. Marzipan (90 g)	120. Breaded products, dumplings (1 serving) 121. Instant soup (1 plate) 122. Mustard (1 teaspoon) 123. Mayonnaise (1 tablespoon, 20 g) 124. Ketchup (1 teaspoon) 125. Spices: pepper, paprika (1 pinch) 126. Salt (1 pinch) 127. Jam (1 teaspoon) 128. Sugar (1 teaspoon) 129. Honey (1 teaspoon) 130. Snack products: chips, peanuts, popcorn, etc. (1 bag, 50 g) 131. Other frequently consumed foods: 131.1 131.2	132. Sugary carbonated drinks: Coca-Cola, Fanta, tonic, etc. (1 can) 133. Low-energy (zero, light) carbonated drinks (1 can) 134. Freshly squeezed orange juice (1 glass, 200 mL) 135. Other freshly squeezed fruit juice (1 glass, 200 mL) 136. Commercial fruit juices/nectars (1 glass, 200 mL) 137. Decaffeinated coffee (1 cup) 138. Espresso (1 cup, 50 mL) 139. Tea (1 cup) 140. Must (1 cup) 141. Rosé wine, glass (100 mL) 142. Dessert wine, glass (100 mL) 143. Young red wine, glass (100 mL) 144. Aged red wine, glass (100 mL) 145. White wine, glass (100 mL) 146. Sparkling wine, glass (100 mL) 147. Beer (1 mug, 330 mL) 148. Liqueur (1 glass, 50 mL) 149. Distilled spirits: brandy. Whiskey, vodka, gin, cognac (1 shot, 50 mL)

**Table 2:** Sociodemographic characteristics of the sample (*n* = 93).

	*n* (%)
Sex	
Men	39 (41.9%)
Women	54 (58.1%)
	mean (±SD)
Age (years)	24.3 (±2.6)
Weight (kg)	71.7 (±18.5)
Height (m)	1.72 (0.2)
BMI (kg/m^2^)	23.6 (±4.0)
	*n* (%)
Underweight (<18.5)	7 (7.5)
Normal weight (18.5–24.9)	51 (54.8)
Overweight (25–29.9)	29 (31.2)
Obesity (≥30)	6 (6.5)

Abbreviations: BMI: Body Mass Index. Categorical variables are shown in sample size and percentage, and continuous variables by mean and standard deviation (SD).

**Table 3:** Mean daily dietary intakes estimated via FFQ and 24 hR (*n* = 93).

Nutrient	FFQ Mean (± SD)	24 hR * Mean (±SD)
Energy (kcal)	2824.5 (1347.3)	2288.4 (554.1)
Water (ml)	1361.3 (329.3)	1041.3 (474.0)
Protein (g)	114.4 (44.4)	102.5 (29.1)
Carbohydrates (g)	294.7 (156.9)	228.8 (72.6)
Fiber (g)	33.2 (17.5)	21.6 (9.1)
Fat (g)	121.5 (67.4)	103.9 (31.4)
Saturated (g)	43.1 (24.8)	37.5 (13.2)
Monounsaturated (g)	44.8 (26.8)	34.3 (11.1)
Polyunsaturated (g)	20.7 (12.1)	22.9 (10.2)
Cholesterol (mg)	468.6 (241.6)	479.1 (217.4)
Calcium (mg)	1055.8 (475.1)	883.3 (367.3)
Sodium (mg)	3134.3 (1679.1)	2523.7 (961.8)
Phosphorous (mg)	1947.3 (783.0)	1543.6 (496.6)
Vitamin B_12_ (µg)	8.4 (4.3)	8.1 (5.3)
Vitamin C (mg)	207.5 (135.3)	99.7 (67.4)
Vitamin D (µg)	5.5 (4.1)	5.9 (6.5)

FFQ, Food frequency questionnaire; 24 hR, 24 h dietary recall. * 24 hR are average of 3 × 24 h recalls.

**Table 4:** Validation of FFQ (*n* = 93 ^a^).

Nutrient	Validation FFQ vs. 24 hR			
	Pearson Correlation Coefficient		ICC	
	Unadjusted ^b^	Energy-Adjusted ^c^	Unadjusted	Energy-Adjusted
Energy (kcal)	0.24 *		0.13 *	
Water (ml)	0.13	0.07	0.84	0.13
Protein (g)	0.08	0.11	0.09	0.21
Carbohydrates (g)	0.32 **	0.23 *	0.34 **	0.37 *
Fiber (g)	0.08	0.14	0.01	0.25
Fat (g)	0.14	0.01	0.09	0.01
Saturated (g)	0.11	0.04	0.15	0.08
Monounsaturated (g)	0.16	0.16	0.24	0.28
Polyunsaturated (g)	0.11	0.04	0.13	0.08
Cholesterol (mg)	0.26 *	0.33 *	0.22 **	0.50 **
Calcium (mg)	0.07	0.08	0.07	0.15
Sodium (mg)	0.22 *	−0.08	0.01	−0.17
Phosphorous (mg)	0.05	0.01	0.05	0.01
Vitamin B_12_ (µg)	0.25 *	0.23 *	0.38 *	0.37 *
Vitamin C (mg)	0.19	0.22 *	0.25 *	0.36 *
Vitamin D (µg)	0.17	0.24 *	0.29	0.49 *

FFQ, food frequency questionnaires; ICC, intraclass correlation coefficient. ^a^ Intakes were log10 transformed to improve normality. ^b^ Unadjusted for energy. ^c^ Adjusted for energy. * *p* < 0.05; ** *p* < 0.01.

**Table 5:** Classification (% of participants), of energy, water, fiber, and macronutrient distribution in opposite quintiles and the same/adjacent quintile in FFQ vs. 24 hR * (*n* = 93).

Nutrients	Q1-24 hR and Q5-FFQ2	Q5-24 hR and Q1-FFQ	Classified in FFQ within 1 Quintile in 24 hR
Energy (kcal)	3.2	4.3	57.1
Water (ml)	3.2	3.2	55.9
Protein (g)	5.3	3.2	58.1
Carbohydrates (g)	2.1	1.1	66.6
Fiber (g)	5.3	5.3	49.4
Fat (g)	0.0	6.4	58.1

FFQ, food frequency questionnaire; 24 hR, 24 h dietary recall. * 24 hR are average of 3 × 24 hR.

## Data Availability

There are restrictions on the availability of the data of this trial due to the signed consent agreements around data sharing, which only allow access to external researchers for studies following the project’s purposes. Requestors wishing to access the trial data used in this study can make a request by emailing pep.tur@uib.es.

## Supplementary Materials

The following supporting information can be downloaded at: https://www.mdpi.com/article/10.3390/nu16152490/s1.
