# Supplementary material for: Development and Evaluation of the Reliability of a Semi-Quantitative Food Frequency Questionnaire to Assess the Intake in the Serbian Population
Source: Nutrients. 2024 Jul 31;16(15):2490. doi: 10.3390/nu16152490 (PMC11313923; doi:10.3390/nu16152490)
Supplement: Supplementary file 1 [file nutrients-16-02490-s001.zip › nutrients-3106590-supplementary.pdf]

ID učesnika:

Datum:

Za svaku namirnicu označite polje koje označava učestalost PROSEČNE KONZUMACIJE TOKOM PROTEKLE GODINE. Moraju se uzeti u obzir letnje/zimske varijacije. Na primer, ako ste konzumirali sladoled 4 puta mesečno, samo tokom 3 letnja meseca, vaša prosečna konzumacija tokom protekle godinu je 1 mesečno (4 puta mesečno x 3 meseca/ 12 meseci = 1 mesečno)

	**NIKADA ILI RETKO**	**MESEČNO**	**NEDELJNO**			**DNEVNO**			
		1 - 3	1	2 - 4	5 - 6	1	2 - 3	4- 5	6+
**I.MLEKO I MLEČNI PROIZVODI**									
1. Punomasno mleko (1 šolja, 200 ml)									
2.Poluobrano mleko (1 šolja, 200 ml)									
3.Obrano mleko (1 šolja, 200 ml)									
4.Biljne zamene za mleko (sojino, ovseno...) (200 ml)									
5.Kajmak (1 supena kašika)									
6.Pavlaka (1/2 šolje)									
7.Milk šejk (1 čaša, 200 ml)									
8.Jogurt (1 čaša, 125 g)									
9. Niskomasni jogurt (1 čaša, 125 g)									
10. Voćni jogurt (1 čaša)									
11.Sveži, sitni sir (1/2 šolje)									
12.Krem sir (1 porcija, 25 g)									
13.Tvrdi/polutvrdi sir (50 g)									
14.Beli sir (50 g)									
15.Krem, puding (1 komad, 130 ml)									
16.Sladoled (1 komad)									
**II. JAJA, MESO, RIBA (**1 PORCIJA ILI 100-150 g, IZUZEV KADA JE DRUGA KOLIČINA OZNAČENA**)**									
17.Jaje (1)									
18.Piletina ili ćuretina, sa kožom (1 porcija ili parče)									
19.Piletina ili ćuretina, bez kože (1 porcija ili parče)									
20.Teletina/govedina (1 porcija)									
21.Svinjetina (1 porcija)									
22.Jagnjetina (1 porcija)									
23.Zečetina (1 porcija)									
24.Džigerica (1 porcija)									
25.Druge iznutrice (1 porcija)									
26.Pršuta (1 parče, 30 g)									
27.Šunka (1 parče, 30 g)									
28.Mesne prerađevine: viršle, kobasice (50 g)									
29.Pašteta (25 g)									
30.Hamburger (1 komad), ćufte (3 komada)									

	NIKADA ILI RETKO	MESEČNO	NEDELJNO			DNEVNO			
		1 - 3	1	2 - 4	5 - 6	1	2 - 3	4 - 5	6+
31.Slanina, pančeta (50 g)									
32. Bela riba: oslić, orada, škarpina, list itd. (1 komad ili porcija)									
33.Plava riba: sardine, tuna, skuša, losos (1 komad ili porcija)									
34.Usoljena riba: bakalar, dimljeni šaran (1 porcija, 60 g)									
35.Ostrige, školjke, dagnje i slično (6 komada)									
36.Lignje, hobotnica (1 porcija, 200 g)									
37.Šrimpovi (4-5 komada, 200g)									
38.Konzervirana riba u salamuri: sardina, tuna...(1 konzerva)									
39.Konzervirana riba u ulju: sardine, tuna... (1 konzerva)									
**III.POVRĆE** (1 PORCIJA ILI 200 g, IZUZEV KADA JE DRUGA KOLIČINA OZNAČENA)									
40.Blitva, spanać									
41.Kupus, karfiol, brokoli									
42.Zelena salata (100 g)									
43.Svež paradajz (1 komad, 150 g)									
44.Šargarepa, bundeva (100 g)									
45.Boranija (zelena, žuta)									
46.Patlidžan, tikvice, krastavci									
47.Paprika (150g)									
48.Špargla									
49. Sok od paradajza (1 čaša, 200 g)									
50.Drugo povrće (artičoka, praziluk, celer)									
51.Crveni luk (pola glavice, 50 g)									
52.Beli luk (1 čen)									
53.Peršun, majkina dušica, origano, itd. (1 prstohvat)									
54.Smrznuti pomfrit									
55.Domaći pomfrit (1 porcija, 150 g)									
56.Restovani ili kuvani krompir									
57. Šampinjoni, lisičarke, druge pečurke									
**IV.VOĆE (1 KOMAD ILI PORCIJA)**									
58.Pomorandža (1 komad), grejpfrut (1 komad) ili mandarine (2 komada)									
59.Banana (1 komad)									
60.Jabuka, kruška (1 komad)									
61.Jagode (6 komada, 1 desertni tanjir)									
62.Trešnje,višnje,šljive (1 šaka/desertni tanjir)									
63.Breskva, nektarina, kajsija (1 komad)									

	NIKADA ILI RETKO	MESEČNO	NEDELJNO			DNEVNO			
		1 - 3	1	2 - 4	5 - 6	1	2 - 3	4 - 5	6+
64. Lubenica (1 kriška, 200-250g)									
65.Dinja (1 kriška, 200-250 g)									
66.Kivi (1 komad, 100 g)									
67.Grožđe (1/2 grozd)									
68.Tropsko voće: ananas, papaja, mango (150-200 g)									
69.Bobičasto voće: borovnice, kupine, maline (100 g)									
70.Avokado (150-200g)									
71.Masline (10 komada)									
72.Kandirano voće (2 komada)									
73.Datule, suve smokve, suvo grožđe, suve šljive (50 g)									
74.Bademi (30 g)									
75.Pistaći (30 g)									
76.Orasi (30g)									
77.Ostalo jezgrasto voće (30 g)									
78.Koliko puta nedeljno imate voće kao dezert?									
**V.ŽITARICE I LEGUMINOZE (**1 porcija)									
79.Sočivo (1 tanjir, 150 g)									
80.Pasulj (1 tanjir, 150 g)									
81.Leblebija (1 tanjir, 150 g)									
82.Grašak (1 tanjir, 150 g)									
83.Beli pšenični hleb (1 kriška, 75 g)									
84.Integralni pšenični hleb (1 kriška, 75 g)									
85.Hleb od drugih žitarica: raž, kukuruz, tortilje itd (1 kriška, 75 g)									
86.Instant žita za doručak (30 g)									
87.Žitarice punog zrna: musli, ovsene pahuljice (30 g)									
88.Pirinač (60 g)									
89.Integralni pirinač (60 g)									
90.Testenina: nudle, makarone, spagete (60 g)									
91.Integralna testenina: nudle, makarone, spageti (60 g)									
92.Pizza (1 parče, 200 g)									
Koliko često konzumirate?									
93.Prženu hranu kod kuće									
94.Prženu hranu u restoranu									
**VI.MASTI I ULJA** (1 supena kašika ili pojedinačna porcija (za prženje, mazanje, za salate, ukupno)									
95.Maslinovo ulje (1 supena kašika)									

	NIKADA ILI RETKO	MESEČNO	NEDELJNO			DNEVNO			
		1 - 3	1	2 - 4	5 - 6	1	2 - 3	4 - 5	6+
96.Devičansko ili ekstra devičansko maslinovo ulje (1 supena kašika)									
97.Maslinovo ulje od komine (1 supena kašika)									
98.Kukuruzno ulje (1 supena kašika)									
99.Suncokretovo ulje (1 supena kašika)									
100.Sojino ulje (1 supena kašika)									
101.Mešavine biljnih ulja (1 supena kašika)									
102.Margarin (porcija, 12 g)									
103.Slani buter (porcija, 12 g)									
104.Neslani buter (porcija, 12g)									
105.Svinjska mast (10 g)									
106.Brend maslinovog ulja koje obično koristite:									
**VII.SLATKA PECIVA I KONDITORSKI PROIZVODI**									
107.Plazma keks (6 komada, 50 g)									
108.Integralni keks (4-6 komada, 50 g)									
109.Čokoladni keks (4 komada, 50 g)									
110.Domaći kolač (50 g)									
111.Kroasan (1 komad, 50 g)									
112.Krofna (1 komad)									
113.Mafini (1-2 komada)									
114.Domaća torta (1 komad)									
115.Uštipci, prženo testo (1 porcija, 100 g)									
116.Čokolada i konditorski proizvodi (30 g)									
117.Kakao (1 kafena kašika)									
118.Bonžita (1 komad)									
119.Marcipan (90 g)									
**VIII.RAZNO**									
120.Panirani proizvodi, knedle (1 porcija)									
121.Supa iz kesice (1 tanjir)									
122.Senf (1 kafena kašika)									
123.Majonez (1 supena kašika, 20 g)									
124.Kečap (1 kafena kašika)									
125.Začini: biber, paprika (1 prstohvat)									
126.So (1 prstohvat)									
127.Džem (1 kafena kašika)									
128.Šećer (1 kafena kašika)									
129.Med (1 kafena kašika)									
130.Snek proizvodi: čips, smoki, kokice, itd (1 kesica, 50g)									
131.Druge često konzumirane namirnice:									
131.1									

	NIKADA ILI RETKO	MESEČNO	NEDELJNO			DNEVNO			
		1 - 3	1	2 - 4	5 - 6	1	2 - 3	4 - 5	6+
131.2									
**IX.NAPICI**									
132.Gazirana pića sa šećerom: coca cola, fanta, tonik... (1 limenka)									
133.Nisko energetska (zero, light) gazirana pića (1 limenka)									
134.Ceđena pomorandža (1 čaša, 200 ml)									
135.Drugo ceđeno voće (1 čaša, 200 ml)									
136.Komercijalni voćni sokovi/nektari (1 čaša, 200 ml)									
137.Dekafeinizirana kafa (1 šolja)									
138.Espreso (1 šoljica, 50 ml)									
139.Čaj (1 šolja)									
140.Šira (100 ml)									
141.Rose vino, čaša (100 ml)									
142.Desertno vino, čaša (50 ml)									
143.Crveno vino, mlado, čaša (100 ml)									
144.Crveno vino, odležano, čaša (100 ml)									
145.Belo vino, čaša (100 ml)									
146. Penušavo vino, čaša (100 ml)									
147.Pivo (1 krigla, 330 ml)									
148.Liker (1 čaša, 50 ml)									
149.Destilovana alkoholna pića: rakija, viski, vodka, džin, konjak (1 čašica, 50 ml)									
150. Koliko godina ste imali kada ste prvi put konzumirali alkoholno piće (vino, pivo ili žestoko piće)?									
151. Koliko godina unazad pijete alkohol redovno?									
